# Investigation into Variation of Endogenous Metabolites in Bone Marrow Cells and Plasma in C3H/He Mice Exposed to Benzene

**DOI:** 10.3390/ijms15034994

**Published:** 2014-03-20

**Authors:** Rongli Sun, Juan Zhang, Lihong Yin, Yuepu Pu

**Affiliations:** Key Laboratory of Environmental Medicine Engineering, Ministry of Education, School of Public Health, Southeast University, Nanjing 210009, Jiangsu, China; E-Mails: sunrongli5318@gmail.com (R.S.); lhyin@seu.edu.cn (L.Y.)

**Keywords:** benzene, endogenous metabolites, bone marrow, plasma, HPLC-TOF-MS

## Abstract

Benzene is identified as a carcinogen. Continued exposure of benzene may eventually lead to damage to the bone marrow, accompanied by pancytopenia, aplastic anemia or leukemia. This paper explores the variations of endogenous metabolites to provide possible clues for the molecular mechanism of benzene-induced hematotoxicity. Liquid chromatography coupled with time of flight-mass spectrometry (LC-TOF-MS) and principal component analysis (PCA) was applied to investigate the variation of endogenous metabolites in bone marrow cells and plasma of male C3H/He mice. The mice were injected subcutaneously with benzene (0, 300, 600 mg/day) once daily for seven days. The body weights, relative organ weights, blood parameters and bone marrow smears were also analyzed. The results indicated that benzene caused disturbances in the metabolism of oxidation of fatty acids and essential amino acids (lysine, phenylalanine and tyrosine) in bone marrow cells. Moreover, fatty acid oxidation was also disturbed in plasma and thus might be a common disturbed metabolic pathway induced by benzene in multiple organs. This study aims to investigate the underlying molecular mechanisms involved in benzene hematotoxicity, especially in bone marrow cells.

## 1. Introduction

Benzene is an important industrial chemical widely used in the production of many products and is also a component of cigarette smoke, gasoline, crude oil and automobile emissions [1]. The hematopoietic system is the most critically affected target tissue following exposure to benzene in humans and animals. Benzene is an established cause of acute myeloid leukemia (AML), myelodysplastic syndromes (MDS), and very likely also lymphocytic leukemias and non-Hodgkin lymphoma (NHL) in humans [2–4]. Benzene was identified as an environmental carcinogen in 1982 [5] and placed in the Group 1 human carcinogen category in 1987 by the International Agency for Research on Cancer [6].

The major adverse health effect from exposure to benzene is hematotoxicity. Benzene can cause a decrease in the three major circulating cell types: platelets (thrombocytopenia), red blood cells (anemia) and white blood cells (leukopenia); and an increase in red cell mean corpuscular volume [7,8]. Sustained exposure may result in continued marrow depression involving multiple cell lineages. This multi-lineage depression of blood counts is also known as pancytopenia [9]. Continued exposure may eventually lead to damage to the bone marrow, accompanied by pancytopenia or aplastic anemia. Benzene metabolism is inherently complex [10], and its secondary metabolism occurs in the bone marrow [11,12]. It is clear that bone marrow is the most critical target organ for benzene metabolites, and both progenitor cells and stromal cells in bone marrow have been considered to be potential targets of benzene hematotoxicity. There are a number of mechanistic studies in the literature that can help us to understand the primary mode of action for benzene, and significant progress has been made in this area. These studies describe effects of benzene such as chromosomal aberrations [13], covalent binding [14], and gene mutations [15]; as well as newly identified mechanisms that include alterations in gene expression [16], oxidative stress [17], epigenetic regulation [18], and immune suppression [19]. In addition, a number of biomonitoring studies have estimated internal benzene exposure for humans, and these have identified and quantified benzene or its biological adducts in blood, urine or expired air; urinary biomarkers include S-phenylmercapturic acid (SPMA), trans, trans-muconic acid (ttMA), phenol, catechol and hydroquinone. Although there is a wealth of epidemiologic data regarding benzene in humans and animals, exposure and toxicology data on benzene, and the mechanism of action for the hematotoxic effects of benzene are not completely understood; and assessment of internally based metabolites responsible for these effects are not currently available.

A systems biology approach to disease-related biology is revolutionizing our knowledge of the cellular pathways and gene networks that underlie the onset and progression of disease, and their associated pharmacological treatments [20]. The study of metabonomics depends upon the production of global metabolite profiles that enable diagnostic changes in the concentrations, or proportions, of low-molecular-weight organic metabolites in samples (such as biofluids and organ extracts) to be assayed. Such investigations thus generate metabolic phenotypes (metabotypes [21]), and, by studying these, it may be possible to identify target organ response to a specific toxicant [22], to assess the toxicity of candidate chemical agents, and to gain new insights into the mechanisms of toxicity of xenobiotics [23]. Kristin *et al*. carried out metabolomic analyses of stem cell samples from peripheral blood collected from a cohort of patients before hematopoietic cell transplantation, and the results suggested that the development of therapy-related myelodysplasia syndrome (t-MDS) was associated with dysfunctions in cellular metabolic pathways, including those involved in the metabolism of alanine, aspartate, glyoxylate, dicarboxylate, and phenylalanine; the citrate acid cycle; and aminoacyl-t-RNA biosynthesis [24]. A recent study suggested that the acridone derivative, 2-aminoacetamido-10-(3,5-dimethoxy)-benzyl-9(10*H*)-acridone hydrochloride, altered metabolism of fatty acids, nucleosides, amino acids, glycerophospholipid, and glutathione; it also induced oxidative stress-mediated apoptosis in CCRF-CEM leukemia cells [25]. Metabonomic approaches also enable identification of predictive markers and biomarkers of disease progression. Huang *et al.* used metabonomic profiling to identify a putative specific biomarker pattern in urine as a noninvasive bladder cancer (BC) detection strategy, and found carnitine C9:1 and component I (in a combined biomarker pattern) with a high sensitivity and specificity that allowed discrimination of bladder cancer patients [26]. One of the major analytical techniques used for global metabolic profiling at this time is mass spectrometry. Mass spectrometry (MS) occupies a major role in holistic metabolite profiling due to its sensitivity and widespread availability. Liquid chromatography (LC-MS) is currently the most widely used mass spectrometry technology, especially in the life science and bioanalytical sectors, due to its ability to separate and detect a wide range of molecules [27].

In our previous study [28], the results indicated that pathways of purine, spermidine, fatty acids, tryptophan, and peptide metabolism were disturbed in benzene-exposed mice; but this study was restricted to urine, which meant that relevant information regarding the hematopoietic system and interactions among compartments was lost. Compared with biofluids, bone marrow (as a target organ) more directly reflects the pathophysiologic state of disease processes induced by benzene. Endogenous metabolites in plasma reflects systemic metabolic effects associated with benzene exposure, while bone marrow cell metabolites analyses enable a more precise investigation of local metabolic changes. However, there are presently no studies reported regarding benzene-induced metabolic changes in bone marrow cells or plasma.

In the present study, we used an integrated approach that entailed metabonomic analyses (based upon HPLC-TOF-MS) of bone marrow cells and plasma to discern changes in the respective metabolomes and the interactions between the two compartments. Our aims were to study the variation of endogenous metabolites involved in benzene toxicity from metabonomic information derived from bone marrow, and to identify specific endogenous metabolites in plasma as potential biomarkers of benzene’s toxic hematopoietic effects.

## 2. Results and Discussion

### 2.1. Body Weights and Relative Organ Weights

**Figure 1.**
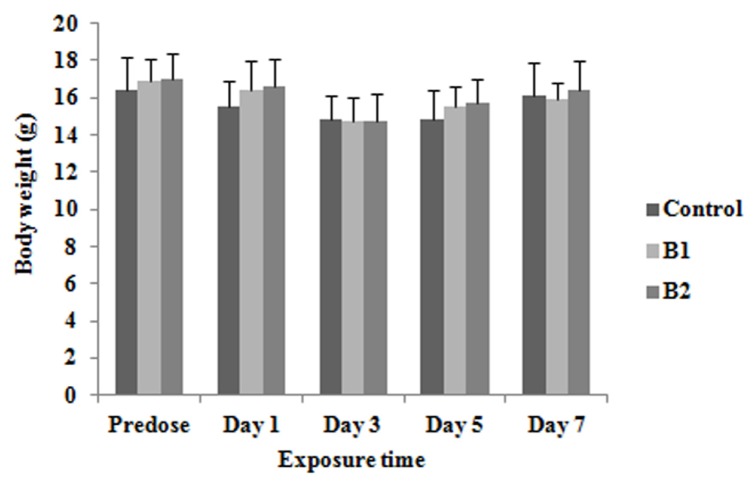
Body weight of mice at each time point and dose. Each bar represents means ± standard deviation (SD) from one-way ANOVA.

The mice in benzene 2 group (receiving 600 mg/kg b.w.) manifested some irritability and lethargy after benzene exposure for seven consecutive days. There were no significant differences in the body weights of mice at any of the time intervals analyzed (Figure 1) (*p* > 0.05), suggesting that the toxicity induced by benzene was insufficient to cause observable body weight changes. The relative organ (liver, spleen, lung, and kidney) weights of mice are presented in Table 1. There was a significant decrease in relative lung weights in benzene 2 group mice on the seventh day of benzene exposure. In addition, there was a significant decrease in relative spleen weights in both benzene 1 and 2 groups.

### 2.2. Blood Parameters and Bone Marrow Smear

**Figure 2.**
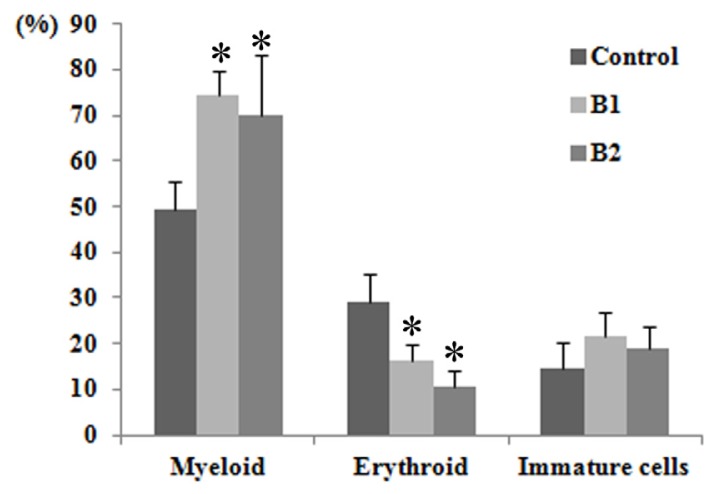
Bone marrow smear examination in male C3H/He mice following 7 days of benzene exposure. * significant difference compared with control group (*p* < 0.05).

The parameters of peripheral blood and bone marrow smears were investigated to assess the hematotoxicity of benzene. As shown in Table 2, a significant decrease in RBC number and hemoglobin (Hgb) concentration occurred in both groups of mice following exposure to benzene for seven days. No effect on WBC number was found, which may be due to the relatively short time of exposure. The platelet (pit) was reduced in the benzene-exposed groups, but not to a statistically significant extent. The bone marrow smears were made to observe the extent of nucleated cell proliferation and cell morphology (Figure 2). The results showed significant myeloid hyperplasia and a marked reduction of erythroidin benzene groups, while no significant difference was observed in the ratio of immature cells in benzene exposure groups. These observations suggest that benzene exposure leads to hematotoxicity.

### 2.3. LC-MS Fingerprinting of Mouse Bone Marrow Cells and Plasma

**Figure 3.**
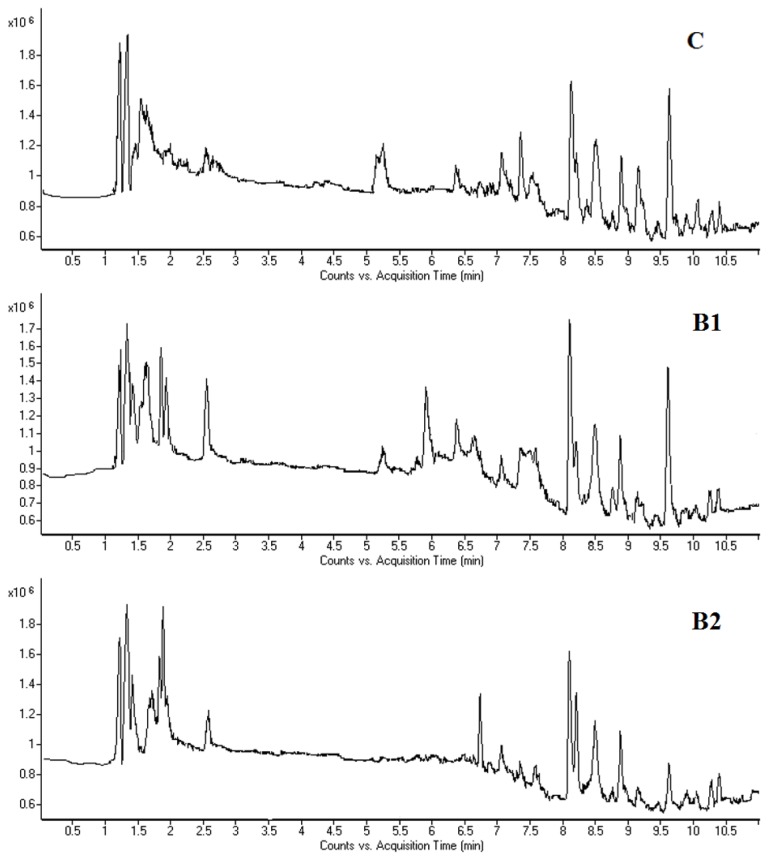
Total ion chromatograms (TICS) of bone marrow cell samples obtained from the control group (**C**), and benzene 1 (**B1**) and benzene 2 groups (**B2**) of male C3H/He mice following 7 days of benzene exposure, using LC/MS (positive mode).

**Figure 4.**
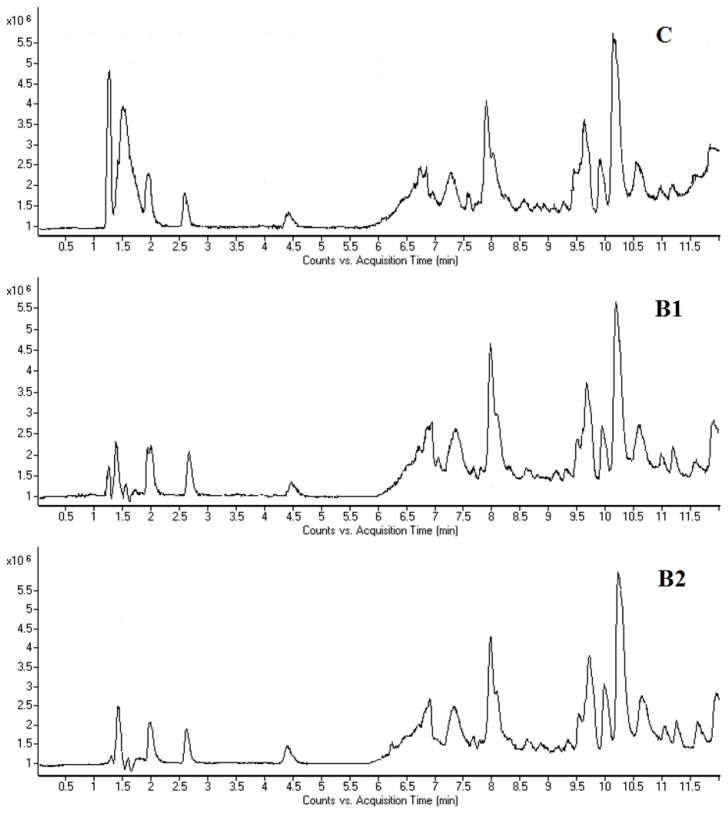
Total ion chromatograms (TICS) of plasma samples obtained from the control group (**C**), and benzene 1 (**B1**) and benzene 2 groups (**B2**) of male C3H/He mice following 7 days of benzene exposure, using LC/MS (positive mode).

Typical HPLC-MS total ion current (TIC) chromatograms of mouse bone marrow cell and plasma samples on day seven taken from the control and benzene-exposed groups are shown in Figures 3 and 4. As shown in Figure 3, there were significant visual differences in the TIC among the groups, especially from 1 to 3 min. The difference between the control group and benzene-exposed groups was more apparent than that between the two dosed groups. A similar metabonomic profile difference was also observed in the TIC of plasma (Figure 4).

### 2.4. Principal Component Analysis and Discovery of Changed Endogenous Metabolites

**Figure 5.**
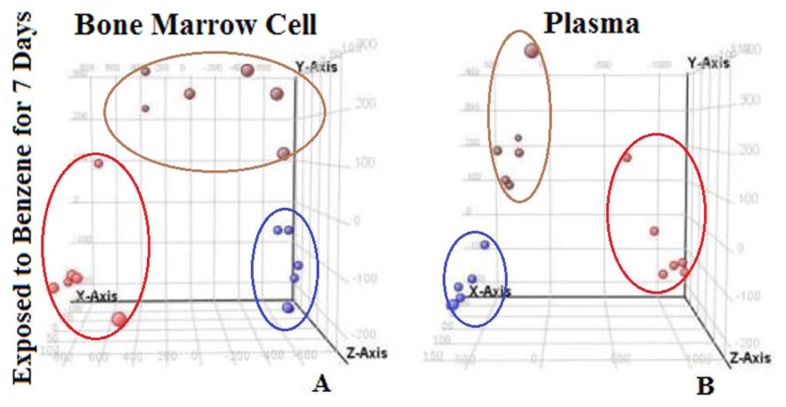
3D PCA score plot of the metabolic profiles of bone marrow cells (**A**) and plasma (**B**) in the control group (red), benzene 1 group (brown), and benzene 2 group (blue) on exposure day 7.

We determined metabolites that were responsible for the changes illustrated above by using one-way ANOVA (*p* < 0.05, fold-change ≥ 2). Principal Component Analysis (PCA) was used to further select biomarkers that could discriminate between groups. PCA (an unsupervised method), is quite useful in distinguishing the several thousand biochemical endpoints retrieved from each sample. In the PCA score plots, each spot represents a metabonomic sample and each assembly of samples expresses a unique metabolic pattern at different time points. This analysis was successful in this experiment because more than 80% of the variability was explained using four components. PCA score plots derived from levels of 16 metabolites from bone marrow cells showed marked differences between the control and benzene-exposed mice on day seven. Similar results can be found in the PCA score map derived from plasma metabolites (Figure 5). The results showed that benzene exposure induced significant changes in 16 metabolites in bone marrow cells and 25 metabolites in plasma, with some metabolites changed in more than one compartment. These metabolites were considered to be potential biomarkers of benzene action.

### 2.5. Identification of Changed Endogenous Metabolites

**Figure 6.**
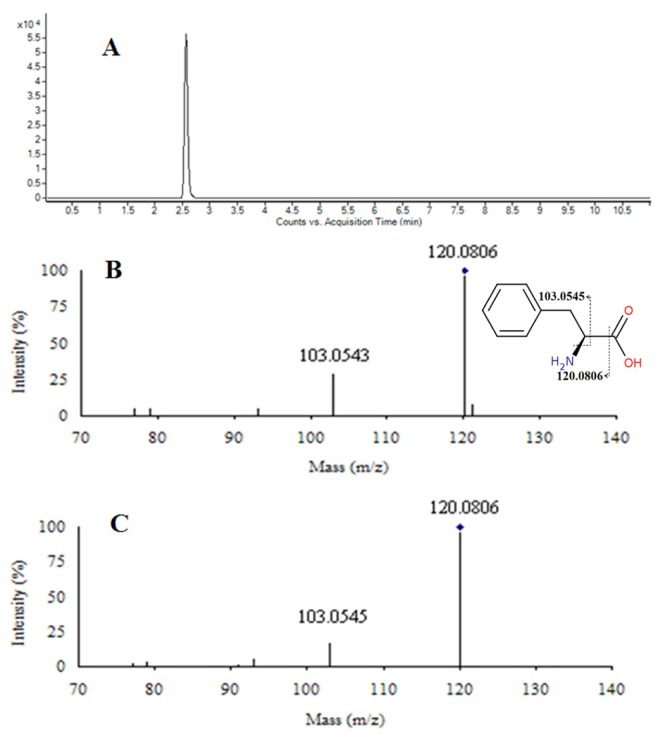
Identification of a selected marker (*m*/*z* 166). (**A**) Extracted ion chromatogram (EIC) of *m*/*z* 166; (**B**) MS/MS spectrum of the ion; (**C**) MS/MS spectrum of a commercial standard l-phenylalanine. The collision energy was 20 V.

Herein, we took the ion at *m*/*z* 166 ([M + H]^+^) as an example to illustrate the identification process. First, the corresponding quasi-molecular ion peak was found according to the retention time in the extracted ion chromatogram (EIC) of *m*/*z* 166 (Figure 6A). The accurate mass of the quasi-molecular ion was found as *m*/*z* 166.0863, and C_9_H_11_NO_2_ was calculated as the most probable molecular formula using Agilent MassHunter software. Then, we conducted its fragmentation by tandem MS. Three major fragment ions were found at *m*/*z* 103.0545 and 120.0806, which represent the fragments of [C_8_H_7_]^+^ and [C_8_H_10_N]^+^, respectively. With the aforementioned information, we searched the freely accessible databases HMDB (http://www.hmdb.ca), METLIN (http://metlin.scripps.edu) and KEGG (http://www.kegg.jp). Finally, considering elemental composition, fragmentation patterns and chromatographic retention behavior, the *m*/*z* of 166 was identified as l-phenylalanine, which was then validated using a standard (Figure 6B,C). Likewise, other biomarkers have been identified and are listed in Table 3. However, the remaining biomarkers (data not shown) were unidentifiable due to insufficient intensity for the MS/MS experiments, or due to restrictions in the current metabolite databases.

### 2.6. Biological Significance of Endogenous Metabolites Alternations in Bone Marrow Cells and Plasma

Metabolic profiling contributes diagnostic information and presents mechanistic insights into the biochemical effects of toxins [29]. The variation of endogenous metabolites in bone marrow may be indicative of benzene’s toxic hematopoietic mechanisms, and the fluctuation may then be observed in plasma. Since blood is circulated around the body, the endogenous metabolites alternations in multi-organs induced by benzene can also be reflected in the blood. This may be helpful in identifying potential biomarkers of toxic effects that would relate to adverse health effects, and that could be monitored in blood. Therefore, in this study, we analyzed benzene-induced endogenous metabolites changes in bone marrow cells and plasma.

#### 2.6.1. Significance of Changed Endogenous Metabolites in Bone Marrow Cells Induced by Benzene

Benzene is hematotoxic and leukemogenic in humans and induces bone marrow suppression in rodents. In recent years, research into the metabolic pathways involved in the renewal and differentiation of HSC and hematopoietic system diseases has intensified. In this study, we uncovered and identified five potential biomarkers in bone marrow cells, and expect to use these to further investigate the hematotoxic mechanisms of benzene. Our results showed in bone marrow cells increases in the levels of phenylalanine and tyrosine, and a decrease in l-lysine, three of the essential amino acids. In addition, a significant lowering of acetylcarnitine was found in bone marrow cells as compared to controls.

A recent study found an increased level of phenylalanine in stem cells from peripheral blood of t-MDS/AML patients, suggesting that there may exist an alteration in mitochondrial activity in these patients relative to controls [24]. In our study, the significant increase in phenylalanine levels in bone marrow cells might then be a manifestation of mitochondrial dysfunction. Phenylalanine is needed for the synthesis of protein, melanin and tyrosine. The increased bone marrow cell levels of phenylalanine may enhance the metabolic pathway from phenylalanine to tyrosine, resulting in a high concentration of bone marrow cell tyrosine. Higher serum levels of phenylalanine and tyrosine were observed in AML patients, which might be the result of enhanced degradation of proteins from the host experiencing a cancerous condition, and these two amino acids are needed for gluconeogenesis and for catabolism to provide intermediates for the tricarboxylic acid cycle (TCA) cycle [30]. It is possible that the toxicity to bone marrow with benzene exposure may be involved in the TCA cycle in mitochondria. The signaling pathway associated with increased phenylalanine and tyrosine may be one of the vital mechanisms intrinsic to benzene-induced hematotoxicity.

l-lysine is an essential amino acid and exerts antifibrinolytic activity by inhibition of fibrinolysis, and exerts a protective effect on platelets [31]. Lysine acetyltransferases were reported to play a key role in leukemogenesis and interact with Runx1 (or AML1), one of the most frequent targets of chromosomal translocations in leukemia [32]. Carnitine is synthesized from lysine residues in existing proteins, and then used to further synthesize acetylcarnitine via carnitine palmitoyl transferase I. Acetylcarnitine is an acetic acid ester of carnitine that facilitates movement of acetyl CoA into the matrices of mammalian mitochondria during the oxidation of fatty acids [33], and it has been observed that lower levels of acetylcarnitine are found in the blood of AML patients [30]. Ito *et al*. showed that fatty acid oxidation was associated with hematopoietic stem cell proliferation and differentiation, which determines whether they undergo symmetric or asymmetric cell division [34]. Therefore, decreases in bone marrow cell lysine and acetylcarnitine levels are likely associated with a down-regulation of carnitine synthesis, which then disturbs oxidation of fatty acids in hematopoietic stem cells that are exposed to benzene. In addition, significant changes in the levels of these compounds in bone marrow were traced in plasma, and we found specific metabolites that related to hematopoietic toxicity of benzene. It is worth noting that only acetylcarnitine levels in plasma decreased, consistent with the changes in bone marrow, indicating that lower levels of acetylcarnitine in plasma might be indicative of hematotoxic effects of benzene.

#### 2.6.2. Significance of Changed Endogenous Metabolites in Plasma Induced by Benzene

The levels of five significantly changed metabolites in bone marrow were also traceable in plasma. Only a reduction in acetylcarnitine was found in plasma. In addition, benzene exposure caused an elevation of l-histidine and pyrrolidone carboxylic acid, concomitant with decreases in 5-hydroxyindoleacetic acid, histamine, *N*-methylhistamine, and palmitoyl-l-carnitine in plasma (Table 3). These significantly changed endogenous metabolites could be used to illustrate the multiple-organ toxicity induced by benzene.

Benzene was demonstrated to cause a disturbance in histidine-related metabolism, including a significant decline in histamine and *N*-methylhistamine, concomitant with an elevation in histidine. The high plasma histidine in the benzene-exposed groups may be caused by a diminished activity of histidine decarboxylase (HDC) [35], which results in inhibiting decarboxylation of histidine to histamine in benzene-exposed mice. Histamine is an amine derived by enzymatic decarboxylation of histidine, which plays a pivotal role in a number of processes, including inflammation, allergic reactions, gastric acid secretion and neurotransmission [36]. Histamine was reported to inhibit production of reactive oxygen species (ROS) in CML cells via the H_2_-receptor (H_2_R) [37]. A salt of histamine, histamine dihydrochloride (HDC), is used as a drug for the prevention of relapse in patients diagnosed with AML [38,39]. Recently, Aurelius *et al*. found that HDC acted on H_2_R expressed by leukemia cells to reduce ROS formation, which might impact the effectiveness of histamine-based immunotherapy [40]. Phenolic metabolites of benzene accumulate in the bone marrow where myeloperoxidase and other peroxidases convert them to reactive semiquinones and quinines [41], which can further lead to the formation of ROS [42]. Histamine might thereby be consumed in order to respond to ROS generation induced by benzene.

Palmitoylcarnitine is a long-chain acyl fatty acid derivative ester of carnitine that facilitates the transfer of long-chain fatty acids from the cytoplasm into mitochondria during the oxidation of fatty acids. Palmitoylcarnitine was shown to stimulate the activity of caspases 3, 7 and 8, and the level of this long-chain acylcarnitine increased during apoptosis [43]. Ibuki *et al*. reported that benzene metabolites induced an anti-apoptotic effect, and that the effect was mainly due to the production of ROS by benzene metabolites (*p*-benzoquinone and hydroquinone) that inhibited caspase-3 activation. Inhibition of apoptosis, aberrantly prolonging cell survival, may contribute to cancer by facilitating the creation of mutations and by allowing a permissive environment for genetic instability [44]. Thus, the decreased plasma palmitoylcarnitine levels in the benzene-exposed groups may be related to disturbed fatty acid metabolism and the suppression of apoptosis by inhibiting caspase activation.

5-Hydroxyindoleacetic acid (5-HIAA) is a breakdown product of serotonin and levels of these substances may be measured in plasma to monitor progression of diseases such as carcinoid tumors [45] and pulmonary hypertension [46]. In the present study, plasma 5-HIAA was found to be notably reduced in benzene-exposed mice, and this may due to benzene toxicity.

Pyrrolidonecarboxylic acid can be irreversibly converted to glutamate, which is used to generate glutamine. Peng *et al*. [47] investigated amino acid concentrations during induction and preconsolidation therapy in cerebrospinal fluid (CSF) of children with lymphoblastic leukemia (ALL) with or without CNS involvement. However, they did not find any significantly changed amino acid levels except for higher baseline glutamine levels, indicative of a greater risk for CNS leukemia. Although increased levels of glutamine were not detected in the plasma of mice exposed to benzene, the increased pyrrolidonecarboxylic acid might indirectly reflect an early influence of benzene on glutamate metabolism.

## 3. Experimental Section

### 3.1. Chemicals and Reagents

Benzene was purchased from Sigma Co. (St. Louis, MO, USA); and corn oil from COFCO (Beijing, China). Ultrapure water (18.2 MO) was prepared with a Milli-Q water purification system (Millipore, Bedford, MA, USA). LC/MS grade methanol and acetonitrile were purchased from Spain Scharlau, Ltd. (Barcelona, Spain); and analytical grade formic acid was supplied by Dikma Corp. (Richmond, NY, USA).

### 3.2. Ethics Statement

This study was carried out in strict accordance with the recommendations of the Guide for the Care and Use of Laboratory Animals of the State Committee of Science and Technology of the People’s Republic of China. The protocol of experiments was reviewed and approved by the Research Ethics Committee of the Southeast University (approval number: 20130027). Animals were maintained and experiments were conducted in accordance with the Institutional Animal Care and Use Committee of Southeast University.

### 3.3. Animals and Treatments

Eighteen male C3H/He mice (aged 4 weeks, and weighing 17.11 ± 1.03 g) obtained from Wei Tong Li Hua Laboratory Animal Co. Ltd. (Beijing, China) were acclimatized for one week in the Specific-Pathogen Free (SPF) animal facility prior to administration of substances. Animals were maintained under a 12-h light/12-h dark cycle at a temperature of 25 ± 2 °C with a relative humidity of 45%–65%. Animals had *ad libitum* access to a certified standard diet and to drinking water, and were divided randomly into a control group (vehicle, oil; *n* = 6), benzene group 1 (B1: 300 mg/kg b.w.; *n* = 6) and benzene group 2 (B2: 600 mg/kg b.w.; *n* = 6). Mice were injected subcutaneously with either corn oil or a benzene-corn oil mixture once daily for seven consecutive days. The aim of these doses is to examine the corresponding hematotoxicity of benzene. The route of benzene administration (injection, s.c.) was to allow us better control of benzene dosages [48,49].

The body weight of each mouse was recorded every other day during exposure periods. The mice were sacrificed on the seventh day of exposure. The mice were anesthetized with pelltobarbitalum natricum, blood was collected, and then were sacrificed by decapitation. Liver, spleen, lung, and kidneys were excised and weighed. Relative organ weight was calculated as the ratio between organ weight and body weight. Bone marrow cells were flushed from one tibia using a 26-gauge needle to make smears.

### 3.4. Collection of Plasma and Bone Marrow Cell Samples

The plasma and bone marrow cell samples of mice were collected after being exposed to benzene for seven days. Plasma was extracted from whole blood at 3000 rpm for 10 min at room temperature. After acquiring mouse femurs and tibias, the marrow cavities were washed with a 26-gauge needle (on ice). Mouse bone marrow cells were collected after centrifugation at 300× *g* for 10 min at 4 °C. To 5 × 10^6^ bone marrow cells, 1 mL of quenching solution (iced, 0.9% [*w*/*v*] NaCl) was quickly added. All cell samples were centrifuged at 1000× *g* for 1 min. Cell pellets were resuspended in ice-cold 50% aqueous acetonitrile, vortexed, and incubated on ice for 10 min. The extracts were dried in a SpeedVac, and the dried cell extracts were resuspended in 500 μL water. All obtained samples were frozen immediately and stored at −80 °C until analysis.

### 3.5. Sample Preparation and HPLC/MS Analysis

Plasma samples were thawed at room temperature and then centrifuged at 13,000× *g* for 15 min at 4 °C. Each 100 μL aliquot of plasma was mixed with 300 μL of methanol and vortexed to allow for protein precipitation. After centrifugation at 13,000× *g* for 15 min at 4 °C, the combined supernatants were transferred to the auto-sample vials. The bone marrow cells were thawed and could be analyzed directly. For the metabonomic studies, aliquots of 1 μL of each sample were injected into a ZORBAX Eclipse Plus C18 column (3.00 mm × 100 mm × 1.8 μm, Agilent, Santa Clara, CA, USA) using an Agilent 6224 TOF LC-MS system (Agilent). The mobile phase was 0.1% formic acid in water (A) and 0.1% formic acid in acetonitrile (B). The optimized HPLC elution conditions were: (a) plasma: 0–1 min, 5% B; 1–3.5 min, 5%–80% B; 3.5–10 min, 80%–95% B; 10–12 min, 95% B; 12–12.5 min, 5% B; (b) bone marrow cell: 0–0.3 min, 5% B; 0.3–7 min, 5%–95% B; 7–9 min, 95% B; 9–10 min, 5% B. The flow rate was 0.4 mL/min. The column and autosample were maintained at 35 and 4 °C, respectively. The positive ion mode was used for the mass detection. The source parameters were set as follows: drying gas flow rate, 9 L/min; gas temperature, 350 °C; pressure of nebulizer gas, 40 psig; Vcap, 4000 V; fragmentor, 150 V; skimmer, 60 V; and scan range, *m*/*z* 50–1000. The tune mixture solution (Agilent) was employed as the lock mass (*m*/*z* = 121.050873, 922.009798) at a flow rate of 30 μL/min, via a lock spray interface for accurate mass measurement. To confirm the identity of the metabolites obtained after the non-targeted analysis (MS analysis and database search), a LC/6530 Q-TOF-MS (Agilent) was used. The MS/MS analysis was acquired in targeted MS/MS mode with collision energy from 10, 20 and 40 V; and a scan rate of 1 (MS/MS) scans/s.

### 3.6. Data Processing

The Masshunter Data Analysis Software (Ver B.02.01, Agilent Technologies, Barcelona, Spain) was used to analyze results; and the Masshunter Qualitative Analysis Software (Agilent Technologies) was used to obtain the molecular features of the samples, representing different, co-migrating ionic species of a given molecular entity using the Molecular Feature Extractor (MFE) algorithm. Finally, the Masshunter Mass Profiler Professional Software (Ver B.02.02, Agilent Technologies) was used to perform a non-targeted metabolomic analysis of the extracted features. Samples with a minimal absolute abundance of 2000 counts and with a minimum of 2 ions were selected. Multiple charge states were not considered. Compounds from different samples were aligned using a RT window of 0.2% ± 0.15 min and a mass window of 10 ppm ± 2.0 mDa. Only common features (found in at least 75% of the samples of the same condition) were analyzed, correcting for individual bias. Data for PCA analysis were obtained using this software. As a classic unsupervised method (no prior knowledge concerning groups or tendencies within the data sets was necessary) of pattern recognition, PCA was expected to discern through statistical protocols several distinct variables for use as potential biomarkers.

### 3.7. Statistics

Statistical analyses for non-targeted metabonomics analyses were performed using the Mass Profiler Professional Software (Agilent Technologies). The exact masses with significant differences in abundance were determined using a one-way analysis of variance (ANOVA); fold-change >2 was considered to be significant at *p* < 0.05, and was searched against various databases (METLIN, HMDB, LIPID MAPS and KEGG). Otherwise, statistical calculations were performed using the SPSS 15.0 software (SPSS, Chicago, IL, USA). Multiple comparisons were analyzed using one-way ANOVA. Statistical significance was established at a level of *p* < 0.05.

## 4. Conclusions

In conclusion, we applied an LC-MS-based metabonomics approach to investigate benzene-induced toxicity in male C3H/He mice. The combined experimental results of metabonomics, relative organ weights, blood parameters and bone marrow smears indicated benzene-induced hematotoxicity. The obvious metabolic alterations in mouse bone marrow cells and plasma indicated that benzene exposure disrupted metabolism of essential amino acids (lysine, phenylalanine and tyrosine) in bone marrow cells; resulting in benzene-induced hematotoxicity. Benzene also caused disturbance in the metabolism of fatty acids oxidation. The decreased acetylcarnitine in plasma was commensurate with that in bone marrow cells; suggesting that acetylcarnitine in plasma is very likely an appropriate biomarker of benzene hematotoxicity. Our work offers a new clue for further clarification of the mechanism(s) involved in benzene-induced toxicity via studying variation of endogenous metabolites.

## Figures and Tables

**Table 1.** Relative organ weights of male C3H/He mice on benzene exposure day 7.

Group	Relative liver weight	Relative spleen weight	Relative lung weight	Relative kidney weight
Control	6.01 ± 0.32	0.36 ± 0.05	0.69 ± 0.10	1.63 ± 0.14
Benzene 1	6.38 ± 0.31	0.27 ± 0.09 *	0.67 ± 0.08	1.65 ± 0.15
Benzene 2	6.54 ± 0.48	0.25 ± 0.07 *	0.56 ± 0.02 *	1.69 ± 0.06

* significant difference compared with control group (*p* < 0.05).

**Table 2.** Blood parameters in male C3H/He mice on benzene exposure day 7.

Group	WBC (10^9^/L)	RBC (10^12^/L)	Hgb (g/L)	Pit (10^9^/L)
Control	4.05 ± 0.65	7.98 ± 0.39	137.20 ± 5.76	364.25 ± 60.50
Benzene 1	3.87 ± 1.06	7.30 ± 0.14 *	126.67 ± 3.50 *	322.00 ± 107.53
Benzene 2	4.48 ± 0.96	7.32 ± 0.42 *	127.60 ± 7.50 *	282.2 ± 50.01

* significant difference compared with control group (*p* < 0.05).

**Table 3.** Identified endogenous biomarkers in bone marrow cell and plasma on exposure day 7.

Compartment	*m*/*z*	RT (Retention time)	Trend a	*p*-value	Metabolites	Related pathway
Bone Marrow Cell	204.123	1.54	↓	9.30 × 10^−4^	l-Acetylcarnitine	Oxidation of Fatty Acids
	165.0546	1.681	↑	5.14 × 10^−3^	*p*-Coumaric acid	Unknown
	182.081	1.668	↑	3.97 × 10^−4^	l-tyrosine	Tyrosine/Phenylalanine and Tyrosine Metabolism, Catecholamine Biosynthesis
	166.0863	2.548	↑	1.62 × 10^−3^	l-Phenylalanine	Phenylalanine and Tyrosine Metabolism
	147.1168	9.205	↓	1.65 × 10^−4^	Lysine	Lysine Degradation, Biotin Metabolism, Carnitine Synthesis

Plasma	192.0644	1.2775	↓	1.92 × 10^−6^	5-Hydroxyindoleacetic acid	Tryptophan metabolism
	112.0869	1.3	↓	2.06 × 10^−4^	Histamine	Histidine metabolism
	156.0765	1.321	↑	1.08 × 10^−5^	l-Histidine	Histidine Metabolism, Ammonia Recycling Transcription/Translation
	126.1026	1.322	↓	1.32 × 10^−4^	*N*-Methylhistamine	Histidine metabolism
	204.123	1.58	↓	6.01 × 10^−5^	l-Acetylcarnitine	Oxidation of Fatty Acids
	130.0499	2.177	↑	2.88 × 10^−6^	Pyrrolidonecarboxylic acid	Gamma-glutamyl cycle
	400.3421	9.195	↓	3.11 × 10^−7^	Palmitoylcarnitine	Fatty acid Metabolism

a Change trend of benzene exposure mice *vs.* control mice.

Variations compared to control samples: ↑, indicates relative increase in signal; ↓, relative decrease in signal (*p* < 0.05).
